# The prevalence of dementia in a Portuguese community sample: a 10/66 Dementia Research Group study

**DOI:** 10.1186/s12877-017-0647-5

**Published:** 2017-11-07

**Authors:** Manuel Gonçalves-Pereira, Ana Cardoso, Ana Verdelho, Joaquim Alves da Silva, Manuel Caldas de Almeida, Alexandra Fernandes, Cátia Raminhos, Cleusa P. Ferri, A. Matthew Prina, Martin Prince, Miguel Xavier

**Affiliations:** 1 Chronic Diseases Research Center (CEDOC), Nova Medical School | Faculdade de Ciências Médicas, Universidade Nova de Lisboa, Campo Mártires da Pátria 130, 1169-056 Lisbon, Portugal; 2 Department of Neurosciences, H. Santa Maria/CHLN; IMM and ISAMB, Faculty of Medicine, University of Lisbon, Lisbon, Portugal; 3 Champalimaud Neuroscience Programme, Champalimaud Centre for the Unknown, Lisbon, Portugal; 4 Santa Casa da Misericórdia de Mora, União das Misericórdias Portuguesas, Mora, Portugal; 5 USF Fernão Ferro mais, ACES Almada-Seixal, Seixal, Portugal; 6 King’s College London, Centre for Global Mental Health, Health Service and Population Research, Institute of Psychiatry, Psychology & Neuroscience, De Crespigny Park, London, SE5 8AF UK; 7 Universidade Federal de São Paulo, Department of Psychobiology, São Paulo, Brasil

**Keywords:** Dementia, Alzheimer’s disease, Epidemiology, Prevalence, Questionnaires, Cross-cultural psychiatry

## Abstract

**Background:**

Dementia imposes a high burden of disease worldwide. Recent epidemiological studies in European community samples are scarce. In Portugal, community prevalence data is very limited. The 10/66 Dementia Research Group (DRG) population-based research programmes are focused in low and middle income countries, where the assessments proved to be culture and education fair. We applied the 10/66 DRG prevalence survey methodology in Portugal, where levels of illiteracy in older populations are still high.

**Methods:**

A cross-sectional comprehensive one-phase survey was conducted of all residents aged 65 and over of two geographically defined catchment areas in Southern Portugal (one urban and one rural site). Nursing home residents were not included in the present study. Standardized 10/66 DRG assessments include a cognitive module, an informant interview and the Geriatric Mental State-AGECAT, providing data on dementia diagnosis and subtypes, mental disorders including depression, physical health, anthropometry, demographics, disability/functioning, health service utilization, care arrangements and caregiver strain.

**Results:**

We interviewed 1405 old age participants (mean age 74.9, SD = 6.7 years; 55.5% women) after 313 (18.2%) refusals to participate.

The prevalence rate for dementia in community-dwellers was 9.23% (95% CI 7.80–10.90) using the 10/66 DRG algorithm and 3.65% (95% CI 2.97–4.97) using DSM-IV criteria. Pure Alzheimer’s disease was the most prevalent dementia subtype (41.9%). The prevalence of dementia was strongly age-dependent for both criteria, but there was no association with sex.

**Conclusions:**

Dementia prevalence was higher than previously reported in Portugal. The discrepancy between prevalence according to the 10/66 DRG algorithm and the DSM-IV criteria is consistent with that observed in less developed countries; this suggests potential underestimation using the latter approach, although relative validity of these two approaches remains to be confirmed in the European context. We improved the evidence base to raise awareness and empower advocacy about dementia in Portugal, so that the complex needs of frail older people may be met in better ways.

## Background

### Dementia as a public health priority in older people

Recent decades have seen rapid demographic ageing with numbers of people with dementia growing quickly worldwide [1, 2]. Most recent regional estimates of the age-standardized prevalence of dementia in people aged 60 years and over fall between 5.6 and 7.6%. Although dementia was traditionally viewed as more prevalent in developed countries, these estimates range from 4.6% in Central Europe to 8.7% in North Africa and the Middle East [1].

Dementia has become a health and social care priority in many high-income countries, where, on the other hand, epidemiological evidence is becoming increasingly out of date [1]. Findings from some recent European studies [3, 4] have stimulated a renewed debate on secular trends for dementia prevalence [5–7]. It has been recommended that all countries should monitor trends regarding dementia prevalence and burden of disease, through nationally representative surveys to be regularly repeated [1, 2]. This particularly applies to Portugal, where epidemiological data regarding cognitive impairment and dementia are scarce.

### The situation in Portugal

Portugal, a south European country, is still a step behind most developed western countries concerning some sociocultural indicators (as reflected by an illiteracy rate of 5.2% in 2011) [8]. The proportion of the Portuguese population aged 65 years and older has grown from 16.4% in 2001 to 19.0% in 2011 [8]. This age group still has a relatively low educational level (illiteracy rate of 19.5% in 2011) [8] and many will have lived under conditions of economic adversity and undernutrition in childhood, possibly increasing dementia risk.

Portuguese epidemiological studies in the field of cognitive impairment are easy to summarize. In the early 1990’s, the EURODEM group were not able to include any studies from Portugal when reporting only trivial differences in the age-specific prevalence of dementia and Alzheimer’s disease (AD) between participating European countries [9]. In 1994, Garcia et al. estimated the number of Portuguese people with dementia (92,470) and AD (48,706), based on European community prevalence rates [10]. These figures were updated by Eurocode to 153,000 and 90,000 [11], and more recently by Santana et al. [12] (in those of 60 years or over) to 160,287 and between 80,144–112,201, respectively.

Nunes et al. conducted the first Portuguese true epidemiological study of neurocognitive disorders, the POLSCI study [13]. In northern Portugal, they reported prevalences of 2.7% (CI 95% 1.9–3.8%) for DSM-IV dementia, with similar proportions of AD and vascular dementia subtypes, and 12.3% (CI95% 10.4–14.4%) for cognitive impairment ‘no dementia’. However, the sample was not nationally representative. This and other methodological limitations preclude the generalization of POLSCI results to older populations in Portugal. On the other hand, the first national psychiatric epidemiological survey in Portugal unfortunately did not address dementia [14, 15].

### The 10/66 Dementia Research Group studies

The 10/66 Dementia Research Group (10/66 DRG) was formed in 1998 to address the lack of dementia-related epidemiological research in low and middle income countries by: 1) devising a culture and education-fair diagnosis of dementia (the 10/66 DRG dementia diagnostic algorithm – henceforward referred to as ‘10/66 dementia’); 2) carrying out population-based surveys of the prevalence, incidence and impact of dementia in nine low and middle income countries (China, Cuba, Dominican Republic, India, Nigeria, Mexico, Peru, Puerto Rico, Venezuela) [16–19]. The 10/66 DRG protocols for prevalence and incidence surveys, together with a case-finder approach and a brief caregiver psychoeducational intervention, are detailed elsewhere [17]. The 10/66 DRG is committed to create an evidence base to empower advocacy, raise awareness about dementia, and ensure that the health and social care needs of older people are anticipated and met [17].

The implementation in Portugal of the 10/66 DRG protocols, beginning with the prevalence study, provided an opportunity to partially overcome the acknowledged insufficiencies of research in the field [20]. First, this is a fully structured approach, with a fixed diagnostic algorithm [17], lending itself to reassessments of prevalence over time, free of the bias of ever changing diagnostic practices. Second, the method provides comprehensive assessments of sociodemographic characteristics, mental disorders (including depression), physical health, anthropometry, non-communicable disease risk factors, disability/functioning, use of services, care arrangements and caregiver strain [17]. Third, there are concerns that the use of DSM-IV criteria may be systematically underestimating true prevalence in low education, low awareness settings (of which rural Portugal may still be an example). Finally, although the 10/66 DRG diagnostic algorithm has not yet been formally validated in a European context, there is robust evidence of its validity aside from the original validation study [21]: in Cuba (criterion validity against local specialist clinicians’ diagnoses) [18]; in Chennai, India (predictive validity) [22]; in Lebanon (including in persons with mild to moderate dementia) [23]; and also in Singapore (a high income country) [24].

### Aims of the study

We aimed to conduct a prevalence study of neuropsychiatric conditions in Portuguese older people, using the 10/66 DRG protocols. This was an opportunity to implement the validated 10/66 protocols for the first time in a European country, while estimating the prevalence of dementia. Data on depression, overall disability, caregiver strain and arrangements, along with service use, will be reported subsequently.

## Methods

### Study design

We applied the aforementioned 10/66 DRG guidelines for the dementia prevalence studies [17, 20]. A standardized operating procedures manual, covering every aspect of the training and field procedures, is available at http://www.alz.co.uk/1066/.

### Setting

Two catchment areas were mapped, one in Fernão Ferro (Seixal, Setúbal), the other in Mora/Cabeção (Mora, Évora). Both locations are in the south of Portugal. Fernão Ferro, near Lisbon, is considered an urban setting, with predominately lower and middle class residents. Total population is 17,059 (675.3 inhabitants/km^2^; 50.9% female; 18.9% above 64 years; 3.5% illiterate) [8, 25]. Mora/Cabeção, near Évora, includes a small town in the middle of a rural area. Total population is 3595 (21.2 inhabitants/km^2^; 52.2% female; 32.4% above 64 years; 14.4% illiterate) [8, 25]. In this paper, we will address the two catchment areas, mapped in each of the described locations, as ‘urban (Fernão Ferro)’ and ‘rural (Mora)’. Mapping was carried out to identify each distinct household, prior to door-knocking to identify potential eligible participants (aged 65 years and over) with final eligibility confirmed during fieldwork.

### Participants

In each catchment area, all residents aged 65 and over were approached by the research team. The only exclusion criteria were the inability to provide informed consent and the absence of a suitable representative for this purpose. For each participant, a reliable informant was appointed by consensus within each household. In most cases, this was the closest family member and a co-resident, but the main criterion was to choose the person who knew the participant best (being or not a member of the household, or the family). Time spent with the older person was helpful in this decision. Where it was readily apparent that the older person needed care, the main caregiver was selected. Regarding the present study, we do not report on nursing home residents, part of which were also evaluated provided they had been living within the catchment area prior to admission.

### Measures

The full array of measures and corresponding details may be found elsewhere [17, 20]. In short, 10/66 dementia and DSM-IV dementia diagnoses were established from: 1) the Geriatric Mental State (GMS), a semi-structured clinical mental state interview which applies a computer algorithm (AGECAT), identifying organicity (probable dementia), depression, anxiety and psychosis [26]; 2) a cognitive test battery, including the Community Screening Instrument for Dementia (CSI’D’) COGSCORE (which incorporates the CERAD animal naming verbal fluency task) and the modified CERAD 10 word list learning task with delayed recall (for each test, lower scores indicated more cognitive impairment); 3) a brief physical and neurological examination; 4) an informant interview including the structured CSI-D informant interview RELSCORE scale (summarising informant assessment of functional and cognitive decline – lower scores indicating less cognitive impairment) and the modified History and Aetiology Schedule – Dementia Diagnosis and Subtype (HAS-DDS) to establish the onset and course of dementia for subtype determination.

Information on the age and educational level of the participant, and household assets was obtained directly, or from the key informant where information was unreliable. The participant interview also included the World Health Organization Disability Assessment Schedule 2.0 (WHODAS 2.0) [27], and the informant interview included assessment of care arrangements, and the brief version of the Neuropsychiatric Inventory (NPI-Q) [28], assessing both severity of the symptoms and related caregiver distress.

Validated translations were already available for the NPI-Q [29]. All other assessments were carefully translated into European Portuguese, directly from the English originals. Discussions among Portuguese authors (AMC, JAS, MGP and MX) and cross-checking of back-translations with CF and MP all helped to refine the translations. Pilot studies ensured the feasibility of these assessments and provided inputs into the final versions of Portuguese translations.

Dementia diagnosis was made using both 10/66 [17] and DSM-IV research criteria [30]. The corresponding algorithms have been validated and reported elsewhere [18, 31]. Dementia subtypes were also assessed using an algorithm previously described [17], matching the NINDCS-ADRA Alzheimer’s Disease [32], NINDS-AIREN vascular dementia [33] and Lewy Body dementia [34] research diagnostic criteria. All the necessary information for applying this algorithm was retrieved from 10/66 DRG assessments (e.g. HAS-DDS), which also identify other prevalent conditions relevant to the differential diagnosis of dementia and dementia subtype (e.g. psychosis, depression, stroke). Severity of dementia, classified as questionable, mild, moderate or severe, was assessed according to the clinical dementia rating (CDR) [35]. This CDR summary score was calculated through an algorithm using 5-point scales on six domains of cognitive and functional performance (memory, orientation, judgment and problem solving, community affairs, home and hobbies, and personal care).

### Preparation and procedures

There were two fieldwork operational supervisors (AMC, CR) and 14 interviewers, distributed between the two areas. All the interviewers were mental health professionals (psychologists, nurses), acquainted with the study protocol and rigorously trained in the assessment procedures. Training, with focus on inter-rater reliability, was mainly conducted by two psychiatrists (MGP, MX) after attending a one-week meeting for 10/66 DRG researchers run by MP and CF at the Institute of Psychiatry/KCL. Cognitive and neurological/physical assessments were taught by a clinical neurologist (AV). A two-day workshop for the GMS was also conducted in Lisbon by MP, CF, MX and MGP.

Interviews were carried out directly in participant’s own homes or scheduled to primary care or local community facilities, as convenient. All participants received the full assessment, which lasted on average 2–3 h. Sometimes, given the length of evaluation procedures, interviews were split (and assessments completed within no more than one week).

All data were collected directly onto laptop computers using computerized European Portuguese questionnaires driven by Epidata (version 3.1) software. These questionnaires had been developed by the 10/66 DRG, incorporating conditional skips and interactive checking of data consistency. Data were extracted into SPSS® (version 21.0), and cleaning, processing of derived variables, and diagnostic algorithms were done with SPSS syntax files.

### Quality assurance

From the starting of fieldwork, supervisors held monthly meetings with the local team of interviewers and supervised them randomly in the field. Quality control of collected data was assured by reviewing every completed interview, looking for missing data or inconsistencies. A random selection of interviews was thoroughly checked on a monthly basis by the study coordinators, to increase the accuracy of data entry. Data on households, participants and informants were stored in secure repositories, separated from data collected in interviews.

### Statistical analysis

Participant characteristics were presented as frequencies and stratified by study site. Chi-square tests were conducted to assess any demographic differences between the two sites.

Using the same approach of Subramaniam and colleagues [24], the concurrent validity of dementia diagnoses with the DSM-IV and 10/66 criteria was first assessed by comparing the mean cognitive scores, WHODAS 2.0 disability scores, NPI severity and distress scores, and care needs as expressed by caregivers of respondents with no dementia (group 1), those with 10/66 dementia not confirmed by DSM-IV (group 2), and those with DSM-IV dementia (group 3). Differences among the groups were assessed using Chi-square tests for categorical variables, and Scheffe’s test for multiple between group comparisons.

The prevalence of both DSM-IV and 10/66 dementia was estimated separately in urban and rural areas, and stratified by sex and age group. Among dementia cases the distribution of severity and subtype was described.

Finally, the effect of socio-demographic factors (age, sex, education level and number of assets) was modelled using a Poisson regression model, and presented using prevalence ratios.

Data were analysed using the Statistical Package for the Social Science for Windows 21.0 (IBM SPSS, Inc.) and STATA (StataCorp. 2011. Stata Statistical Software: Release 14. College Station, TX: StataCorp LP).

## Results

### General description of the two samples

We completed 1405 interviews with community-dwelling old age participants overall, 697 in the urban area (within Fernão Ferro) and 708 in the rural area (within Mora). In urban Fernão Ferro, 987 potential respondents had been identified in the defined catchment area, but 290 of these (or their representatives) refused to participate, could not be contacted after at least three attempts, or were unable to give informed consent. In rural Mora, 731 potential respondents had been identified but 23 (or their representatives) were not included in this study due to the same reasons. Therefore, participation rates were 81.8% overall, 70.6% in Fernão Ferro and 96.9% in Mora.

Urban participants tended to be younger, better educated, more likely to be married, and less likely to have worked as unskilled manual labourer or in agriculture than their rural counterparts (Table 1). There were no significant differences in gender between the two areas, whereas number of household assets tended to be higher in urban Fernão Ferro. Living alone was more frequent in rural Mora. Pension coverage was high in both regions, even if significantly lower in the urban sample (86.1% vs 94.1%). Income from other sources (paid work, family transfers, rents, others) was uncommon, regardless of the area considered. Full dementia diagnoses could not be attributed to eight individuals due to missing information, hence creating an analysable sample size of 1397.

**Table 1:** Social-demographic characteristics in the urban and rural areas

	Total (*n* = 1405)	Urban (*n* = 697)	Rural (*n* = 708)	χ^2^	*df*	P
Age group (MV)	0	0	0			
65–69 years	359	193(27.7%)	166(23.5%)	28.0	3	<0.001
70–74 years	353	196(28.1%)	157(22.2%)			
75–79 years	340	175(25.1%)	165(23.3%)			
80+ years	353	133(19.1%)	220(31.1%)			
Gender (MV)	0	0	0			
Female	779	373(53.5%)	406(57.3%)	2.1	1	0.149
Educational level (MV)	70	57(8.2%)	13(1.8%)			
No education	225	75(10.8%)	150(21.2%)	124.9	1	<0.001
Primary (not completed)	272	78(11.2%)	194(27.4%)			
Completed primary	770	446(64.0%)	324(45.8%)			
Completed secondary	49	31(4.5%)	18(2.5%)			
Completed tertiary	19	10(1.4%)	9(1.3%)			
Marital status (MV)	7	1	6			
Never married	61	19(2.7%)	42(5.9%)	35.9	4	<0.001
Married or cohabiting	946	509(73.0%)	437(61.7%)			
Widowed	349	140(20.1%)	209(29.5%)			
Divorced or separated	42	28(4.0%)	14(2.0%)			
Previous occupation (MV)	24	27	40			
Professional/managerial	171	127(18.2%)	44(6.2%)	88.2	4	<0.001
Trade/clerical	197	123(17.7%)	74(10.4%)			
Skilled laborer	520	256(36.7%)	264(37.3%)			
Unskilled manual laborer/agricultural worker	497	185(26.5%)	312(44.1%)			
Living arrangements (MV)	0	0	0			
Alone	295	102(14.6%)	193(27.3%)	57.6	4	<0.001
With spouse only	767	388(55.7%)	379(53.5%)			
With adult children	30	25(3.6%)	5(0.7%)			
Other situations	313	182(26.1%)	131(18.1%)			
Source of income (MV)	0	0	0			
Any government or occupational pension	1266	600(86.1%)	666(94.1%)	25.1	1	<0.001
Number of household assets	0	0	0			
0–3 assets	5	2(0.3%)	3(0.4%)	15.1	1	<0.001
4–5 assets	57	14(2.0%)	43(6.2%)			
6–7 assets	1343	681(97.7%)	662(93.5%)			

### Concurrent validation of 10/66 and DSM-IV dementia diagnoses

Results of the concurrent validation of 10/66 DRG and DSM-IV diagnoses are displayed in Table 2. Cognitive function (CSI-D COGSCORE) was significantly higher, and behavioural and psychological symptoms (NPI-Q), informant CSI-D RELSCORE and WHODAS 2.0 disability scores significantly lower in participants not diagnosed with dementia as compared to those with either DSM-IV dementia, or 10/66 dementia not confirmed by DSM-IV criteria. The distribution of these scores was more similar between those with DSM-IV dementia and those with 10/66 dementia not confirmed by DSM-IV, although the CSI-D RELSCORE and NPI-Q severity and carer distress scores were significantly higher in DSM-IV cases. Relevant needs for care were present both in persons with 10/66 and with DSM-IV dementia (58.1% and 79.2%, respectively, compared with 8.2% of those with no dementia). However, caregivers of persons with DSM-IV dementia reported that these participants needed care ‘much of the time’ more often (54.2%) than those of persons with 10/66 dementia (33.3%) (Table 2).

**Table 2:** Concurrent validation of 10/66 and DSM-IV dementia diagnoses

		Group 1: No dementia (*n* = 1268)	Group 2: 10/66 dementia not confirmed by DSM-IV (*n* = 105)	Group 3: DSM-IV dementia (*n* = 24)	Group 1 vs Group 2	Group 1 vs Group 3	Group 2 vs Group 3
CSI’D COGSCORE	Mean (SD)	29.8 (2.5)	20.6 (7.3)	17.5 (7.4)	t value = −9.1 *p* < 0.001	t value = −12.3 *p* < 0.001	t value = −3.1 *p* = 0.061
	Median (IQR)	30.3 (28.4–31.6)	22.1 (18.7–25.3)	20.1 (14.2–21.6)			
CSI’D RELSCORE	Mean	1.3 (1.7)	10.5 (9.1)	20.4 (7.1)	t value = 9.2 *p* < 0.001	t value = 19.1 *p* < 0.001	t value = 9.9 *p* < 0.001
	Median	1 (0–2)	8 (3–16)	21.5 (17–24.4)			
NPI-Q Distress	Mean	1.2 (1.9)	3.3 (3.6)	7.4 (6.0)	t value = 2.1 *p* < 0.001	t value = 6.2 *p* < 0.001	t value = 4.06 *p* < 0.001
	Median	0 (0–2)	2 (0–5)	6 (2–12)			
NPI-Q Severity	Mean	1.4 (2.7)	4.4 (6.1)	10.8 (8.6)	t value = 3.0 *p* < 0.001	t value = 9.3 *p* < 0.001	t value = 6.4 *p* < 0.001
	Median	0 (0–2)	2 (0–7)	8 (3–17)			
WHODAS 2.0 Disability	Mean	16.9 (19.2)	41.1 (30.0)	52.8 (31.9)	t value = 24.2 *p* < 0.001	t value = 35.9 *p* < 0.001	t value = 11.7 *p* = 0.09
	Median	11.1 (0–27.8)	37.5 (15.3-66.7)	55.5 (27.8–80.56)			
Need for care	Needs care much of the time	2.1%	33.3%	54.2%	X^2^ = 381.7 *p* < 0.0001		
	Needs care some of the time	6.1%	24.8%	25.0%			
	No care needed	91.9%	41.9%	21.0%			

Respondents diagnosed with dementia by DSM-IV and also 10/66 diagnoses were defined as ‘DSM-IV dementia’ group (*n* = 24). Respondents not diagnosed with dementia either by DSM-IV or 10/66 diagnoses were defined as ‘No dementia’ group (*n* = 1268). Respondents diagnosed with dementia by 10/66 diagnosis but not confirmed by DSM-IV diagnosis were defined as ‘10/66 dementia not confirmed by DSM-IV’ (*n* = 105) group. *CSI’D COGSCORE* Community Screening Instrument for Dementia Global Cognitive Score, *CSI’D RELSCORE* Community Screening Instrument for Dementia Informant Score, *NPI-Q* Neuropsychiatric Inventory, *WHODAS 2.0* World Health Organization Disability Assessment Schedule

### Prevalence of dementia, and distribution of subtypes and severity among dementia cases

The overall prevalence of dementia was 9.23 (95% CI: 7.80–10.90) according to 10/66 DRG criteria and 3.65 (95% CI: 2.79–4.97) according to DSM-IV criteria (Table 3). The prevalence of 10/66 dementia was similar between the two sites, but in the urban site prevalence of DSM-IV dementia was almost double that in the rural site. Pure AD seemed to be the most prevalent subtype in both areas, especially in the urban area, although around one third of the cases could not be allocated to any particular subtype (Table 4).

**Table 3:** Prevalence of dementia in the two areas (urban and rural) and overall

DSM-IV		65–69	70–74	75–79	80+	Overall
*Urban (n = 690)*	*Female*	#	6.93 (3.33–13.80)	4.60 (1.73–11.63)	11.11 (5.65–20.70)	4.35 (3.06–6.14)
	*Male*	1.18 (0.17–7.90)	2.15 (0.54–8.21)	5.88 (2.47–13.38)	5.08 (1.65–14.64)	
*Rural (n = 707)*	*Female*	#	1.05 (0.15–7.11)	1.16 (0.16–7.82)	6.06 (3.10–11.63)	2.97 (1.95–4.49)
	*Male*	#	#	5.13 (1.93–12.91)	7.95 (3.83–15.79)	
*Overall (n = 1397)*	*Female*	#	4.08 (2.05–7.96)	2.89 (1.21–6.77)	7.84 (4.86–12.41)	3.65 (2.79–4.97)
	*Male*	0.63 (0.09–4.37)	1.29 (0.32–5.02)	5.52 (2.89–10.28)	6.80 (3.69–12.20)	
10/66		65–69	70–74	75–79	80+	Overall
*Urban (n = 690)*	*Female*	0.93 (0.13–6.30)	9.09 (5.40–17.45)	10.34 (5.47–18.72)	25.00 (16.34–36.26)	9.28 (7.24–11.80)
	*Male*	2.35 (0.59–8.94)	3.23 (1.04–9.55)	11.76 (6.44–20.52)	18.64 (10.63–30.64)	
*Rural (n = 707)*	*Female*	#	3.16 (1.02–9.36)	9.30 (4.70–17.57)	16.67 (11.27–23.96)	9.19 (7.29–11.54)
	*Male*	2.74 (0.70–10.15)	9.68 (4.47–19.72)	10.26 (5.20–19.23)	18.19 (11.43–27.69)	
*Overall (n = 1397)*	*Female*	0.50 (0.07–3.50)	6.63 (3.89–11.10)	9.83 (6.19–15.26)	19.61 (14.74–25.61)	9.23 (7.80–10.90)
	*Male*	2.53 (0.96–6.52)	5.81 (3.06–10.75)	11.04 (7.06–16.86)	18.37 (12.89–25.49)	

# no cases; 95% confidence intervals are presented into brackets

**Table 4:** 10/66 dementia subtypes in the two areas (urban and rural) and overall

Dementia Subtype	AREA		
	Urban n (%)	Rural n (%)	Overall n (%)
not allocated	20 (31.3)	19 (29.2)	39 (30.2)
pure Alzheimer’s disease (AD)	31 (48.4)	23 (35.4)	54 (41.9)
pure vascular dementia (VAD)	6 (9.4)	8 (12.3)	14 (10.9)
mixed AD/ VAD	0 (0.0)	7 (10.8)	7 (5.4)
mixed AD/ Dementia with Lewy Bodies (DLB)	5 (7.8)	4 (6.2)	9 (7.0)
Frontal Temporal Dementia (FTD)	2 (2.9)	4 (6.2)	6 (4.7)
TOTAL	64 (100.0)	65 (100.0)	129 (100.0)

CDR clinical severity was generally higher in persons with DSM-IV dementia as compared to those with 10/66 dementia. Questionable diagnoses of DSM-IV dementia were almost inexistent (i.e. one in 51), whereas questionable 10/66 dementia diagnoses represented around one fifth (21.7%) of the total. However, even after excluding all questionable diagnoses, the 10/66 dementia prevalence rate surpassed that of DSM-IV dementia in both areas (Table 5).

**Table 5:** Severity of dementia in the two areas (urban and rural) and overall

		DSM-IV dementia			10/66 DRG dementia		
		Urban	Rural	Overall	Urban	Rural	Overall
	Number of cases	30	21	51	64	65	129
CDR	No dementia	0	0	0	6 (9.4%)	2 (3.1%)	8 (6.2%)
	Questionable	0	1 (4.8%)	1 (2.0%)	13 (20.3%)	15 (23.1%)	28 (21.7%)
	Mild	19 (63.3%)	14 (66.7%)	33 (64.7%)	30 (46.9%)	36 (55.4%)	66 (51.2%)
	Moderate	11 (36.7%)	6 (28.6%)	17 (33.3%)	15 (23.4%)	12 (18.5%)	27 (20.9%)
	10/66 dementia, restricted to CDR mild/moderate/severe				7.53 (5.78–9.78)	8.63 (6.77–10.90)	8.09 (6.77–9.64)

### Correlates of dementia

Table 6 presents data regarding some of the sociodemographic correlates of dementia. The prevalence of dementia was strongly age-dependent for both criteria, and in both sites (Table 3). There was a consistent trend for an inverse association between household assets and the prevalence of dementia. There were also either significant inverse associations between education level and dementia prevalence (urban 10/66 dementia) or trends in that direction (rural 10/66 dementia and urban DSM-IV). However, in the rural area higher education levels were associated with a higher prevalence of dementia. There were no significant associations with sex.

**Table 6:** Prevalence ratios of socio-demographic factors associated within the two areas (urban and rural) and overall

		Urban	Rural	Overall
DSM-IV diagnosis of dementia	Age (per 5-year band)	1.52 (1.11–2.09)	3.12 (1.86–5.23)	1.87 (1.45–2.42)
	Male sex	0.90 (0.41–1.96)	1.44 (0.62–3.38)	1.00 (0.57–1.74)
	Education (per level)	0.71 (0.48–1.03)	1.94 (1.09–3.45)	1.19 (0.82–1.73)
	Assets (per asset)	0.73 (0.47–1.12)	0.61 (0.47–0.78)	0.71 (0.54–0.92)
10/66 DRG diagnosis of dementia	Age (per 5-year band)	1.79 (1.41–2.28)	1.75 (1.35–2.27)	1.74 (1.46–2.07)
	Male sex	1.09 (0.67–1.77)	1.50 (0.95–2.36)	1.24 (0.90–1.72)
	Education (per level)	0.65 (0.51–0.84)	0.87 (0.63–1.22)	0.79 (0.64–0.97)
	Assets (per asset)	0.71 (0.55–0.90)	0.68 (0.58–0.80)	0.72 (0.62–0.83)

## Discussion

We conducted a comprehensive one-phase survey of two catchment areas, one urban and the other rural, in the south of Portugal, with the main objective of estimating the prevalence of dementia. By adopting the 10/66 DRG protocols, we simultaneously diagnosed depression and other psychiatric disorders, which will be reported subsequently. We were interested in the crowded urban/suburban areas near Lisbon (the capital), as well as in the low population-density rural inner country, where risk factors for dementia may differ. One of these latter regions is Alentejo, with a high proportion of older people (24.2%) and educational levels amongst the lowest in Europe (9.6% illiterate) [25]. The choice of our urban and rural catchment areas reflected these interests. In the total community sample, the prevalence of dementia was 9.23% (95% CI: 7.80–10.90) using the 10/66 DRG algorithm and 3.65% (95% CI: 2.79–4.97) using DSM-IV criteria.

### Contextualisation

The final sample size (*n* = 1405) was acceptable, as almost a third of Western European studies report sample sizes under 500 in this sort of studies [1]. In community epidemiological studies, response rates are usually somewhat higher in less developed settings, and those achieved in our study are consistent with, or somewhat better than other recent studies in high income countries [1]. Regarding comparisons between the urban and the rural area, the same research protocol was implemented in both by the same research group, which should warrant a regional comparison on solid grounds. However, potential bias cannot be excluded given higher participation rates in the rural area. In urban Fernão Ferro, a proportion of households turned out to be weekend homes, and weaker social networks perhaps undermined the confidence of potential participants in allowing interviewers to their homes.

We found a higher prevalence of dementia using the 10/66 DRG diagnostic algorithm, compared to the prevalence of DSM-IV dementia, as seen previously across the other 10/66 sites [21]. While the prevalence of 10/66 dementia (9.2%) was aligned within the range reported in other 10/66 DRG studies [21], the prevalence of DSM-IV dementia (3.7%) might be seen as low in the light of recent reviews of the prevalence of dementia by world region [2]. The prevalence of DSM-IV dementia was lower, and the discrepancy between DSM-IV dementia and 10/66 dementia prevalence higher in the rural compared with the urban site. Just as in low and middle income countries, mild dementia may have been under-detected by DSM-IV criteria because of difficulties in establishing the criterion of social impairment, particularly in rural settings characterised by low education and limited awareness of dementia as a condition distinct from normal ageing [21].

Population prevalence may also have been underestimated since this was a study of the community prevalence, excluding formal residential care and nursing home settings. Community formal care is not well developed in Portugal, and most people with severe and advanced dementia would be expected to live in care homes. We did initially attempt to survey residential care and nursing homes located in our catchment areas. Unfortunately we were unable to obtain representative and unbiased estimates of prevalence in this stratum of the population because of problems gaining approval for access to some homes in the urban site that were operating informally without a full licence, and in gaining consent or assent for participation, particularly from those that did not have capacity to consent with no easily accessible nearest relative or legal representative. This may have had an important impact on the overall prevalence of dementia. In 2006 it was estimated that 3.4% of the Portuguese population aged 65 and over lived in care homes [36]; in the catchment areas we surveyed, our initial enumeration of facilities suggested that this proportion may approach 15%, not all of whom would have met prior residence criteria for inclusion in our study.

As reported in many other studies we found that the prevalence of dementia increased with age, and was generally higher among those of lower socioeconomic status (as assessed through household assets). While the prevalence of dementia was also generally higher among those with less education, in the rural site only DSM-IV dementia prevalence but not 10/66 dementia prevalence was higher among those with more education. It is tempting to speculate that this counterintuitive finding may be explained by DSM-IV diagnostic bias operating selectively in low awareness settings.

### Comparison with European and Portuguese studies using other methods

Five western European studies have recently reported a valid comparison of prevalence of dementia over time, but only two (in the UK and Spain) used the GMS as main tool [5], the same standardised psychiatric interview used here as part of the 10/66 DRG protocol. With a one-phase approach, Matthews et al. [3] found a prevalence rate of 6.5%. This was significantly lower than the estimated rate of 8.3%, challenging the idea of an ‘epidemic-like’ projection of dementia rates for the near future in Western countries. In Spain, Lobo et al. found a DSM-IV dementia prevalence of 3.9%, in a two-phase study with case-reassessment by psychiatrists [4]. Regardless of methodological differences, this is close to the DSM-IV dementia prevalence in our study (3.7%), possibly reflecting sociocultural similarities between Spanish and Portuguese populations.

Comparing with the POLSCI study in Northern Portugal [13], we found a slightly higher prevalence of DSM-IV dementia (3.65%, 95% CI: 2.79–4.97) vs 2.7%, 95% CI: 1.9–3.8). In POLSCI, the prevalence of dementia (DSM-IV criteria) was twice as high in the rural sample as compared with the urban one, with the rural/urban prevalence ratio increasing with age. In the present study, while we found that the prevalence of 10/66 dementia was similar in the urban and rural areas, DSM-IV dementia was almost double in the urban sample. There is no straightforward explanation for this contradiction in the results of the two studies, also given the fact that our urban sample tended to be younger and better educated, and the consistency of our findings with 10/66 DRG studies in other settings [21]. Perhaps the differences in response rates between rural and urban areas (which followed the same direction but were larger in our study) may account in part for these discrepancies. In POLSCI, AD and vascular dementia subtypes were similarly frequent among dementia cases, while in our study, although around 30% of cases could not be allocated to any subtype, AD was more frequent than vascular dementia, particularly in the urban area.

Noteworthy, Nunes et al.’s results and ours are not directly comparable: both studies assessed nationally non-representative samples, in different regions of the Country and using different designs and assessments. In POLSCI there was a major interest in minor cognitive impairment, therefore the study did not fully address dementia prevalence and impact. For instance, sample age range (55–79 years) reflected a relatively young group, a limitation acknowledged by the authors [13]. Study design (a primary care-based two-phase survey lacking evaluation of a random sample of negative cases after screening), sample size (*n* = 1146) and overall participation rate (52.6%) may have also influenced POLSCI results. Nevertheless, the overall impression is that prevalence rates for DSM-IV dementia were not strikingly different from those in our study, as confidence intervals widely overlap.

### Limitations and strength of the study

First, by studying these two samples in different catchment areas, we obviously do not claim that the results are generalizable to the whole country. Despite its small territorial size, Portugal is not homogeneous regarding age distribution, literacy, income level, access to health services. However, given the common language and cultural background of the population, the implementation of the same protocol by the same team/supervisors favoured both the internal and external validity of our results.

Second, the criterion validity of 10/66 DRG dementia diagnoses was not established in Portugal by using a gold standard such as an examination by trained clinicians using standardized diagnosis criteria. However, this has been established in a variety of other settings [18, 24, 31], and the concurrent validation we carried out in this study provides further evidence to support construct validity in our context. Analysing predictive validity in our sample would be a complementary approach [19, 22], if feasible in the near future. Third, our inability to access and survey representative samples of older residents of residential care and nursing homes is a significant limitation. We were not able to accurately define the number of older people who could not be approached in institutions. Therefore we did not perform a sensitivity analysis to estimate the number of dementia cases and calculate an adjusted total prevalence in nursing home residents. Hopefully, much can be learnt from our experience, in designing a robust sampling methodology to correct this evidence gap in future national or regional research.

Ours is the first comprehensive one-phase survey of the community prevalence of dementia in Portuguese populations aged 65 and over. By accomplishing this first 10/66 DRG survey in a European country we showed that 10/66 DRG protocols and fieldwork procedures were feasible in this setting. They may be more appropriate for low education European populations such as these Portuguese ones, particularly in rural Mora where nearly half of the older participants had not completed primary education, and where the full validity of standard non-education fair cognitive assessments would be doubtful.

### Implications

Old people with neuropsychiatric problems including dementia and depression represent a frail and important subgroup of the ageing population, whose needs, together with those of their caregivers, are still poorly understood and met. This is especially so in Portugal, were community prevalence studies are crucial, following the priorities set by the National Mental Health Plan 2007–2016 [37]. Only reliable and cross-culturally comparable information about the epidemiology and patterns of care of these conditions may guide adequate disease management [31]: as recently pointed out [7], using a fixed methodology has potential benefits to estimate changes in dementia prevalence [3] or incidence [38] in defined populations, and to foster international comparisons [1].

Regarding this endeavour, the 10/66 DRG protocols for prevalence studies provide robust opportunities as they have now been applied in Europe: 1) they tackle the main neuropsychiatric conditions in old age, dementia and depression; 2) the 10/66 one-stage approach has proven advantages [17] over the two-phase procedures used in most studies on dementia (i.e. screening followed by a diagnostic stage): attrition is marked between the first and other phases; participants with probable dementia are particularly likely to refuse, to move away or to die, leading to informative censoring [39, 40]; 3) catchment area surveys are logistically straightforward and have been favoured by dementia researchers.

## Conclusions

For the first time the 10/66 DRG protocols were successfully implemented in Europe. As in other studies using the same protocol, we report a significant disparity between DSM-IV and 10/66 diagnoses, reinforcing the need to discuss diagnostic algorithms for dementia. Despite need for caution concerning interpretation, the prevalence rate of 10/66 dementia (9.2%) suggests that previous studies possibly underestimated the true dementia prevalence rates in Portugal. Not least, estimates of numbers of Portuguese persons with dementia yielded figures [10–12] that would be clearly exceeded if our findings were nationally generalizable.

Given its ageing population, Portugal is probably about to face a highly demanding health challenge in the coming years, as most of the national health budget is still allocated to hospital acute care instead of directed to chronic disease management. Our results additionally support the need for a new wave of dementia surveys in developed countries.

## Abbreviations

POLSCI: A Portuguese population-based longitudinal study (cf. reference 13)
10/66 DRG: Dementia Research Group
AD: Alzheimer’s disease
AGECAT: The Automated Geriatric Examination for Computer Assisted Taxonomy
CDR: Clinical Dementia Rating
CERAD: Consortium to Establish a Registry for Alzheimer’s Disease
CI: Confidence intervals
CSI’D’ COGSCORE: Community Screening Instrument for Dementia, cognitive score
CSI’D’ RELSCORE: Community Screening Instrument for Dementia, informant score
DSM-IV: Diagnostic and Statistical Manual of Mental Disorders, fourth edition
EURODEM: European Community Concerted Action on Epidemiology and Prevention of Dementia
GMS: Geriatric Mental State examination
HAS-DDS: History and Aetiology Schedule – Dementia Diagnosis and Subtype
NINDCS-ADRA: National Institute of Neurological and Communicative Disorders and Stroke and the Alzheimer Disease and Related Disorders Association Criteria for Alzheimer Disease
NINDS-AIREN vascular dementia: National Institute of Neurological Disorders and Stroke and Association Internationale pour la Recherche et l’Enseignement en Neurosciences
NPI-Q: Neuropsychiatric Inventory – Questionnaire, brief version
WHODAS 2.0: World Health Organization Disability Assessment Schedule version 2.0
